# A comprehensive analysis of rare genetic variation in amyotrophic lateral sclerosis in the UK

**DOI:** 10.1093/brain/awx082

**Published:** 2017-04-18

**Authors:** Sarah Morgan, Aleksey Shatunov, William Sproviero, Ashley R. Jones, Maryam Shoai, Deborah Hughes, Ahmad Al Khleifat, Andrea Malaspina, Karen E. Morrison, Pamela J. Shaw, Christopher E. Shaw, Katie Sidle, Richard W. Orrell, Pietro Fratta, John Hardy, Alan Pittman, Ammar Al-Chalabi

**Affiliations:** 1 Department of Molecular Neuroscience, UCL, Institute of Neurology, Queen Square, London, WC1N 3BG, UK; 2 Department of Basic and Clinical Neuroscience, Maurice Wohl Clinical Neuroscience Institute, King’s College London, SE5 9RX, UK; 3 Centre for Neuroscience and Trauma, Blizard Institute, Queen Mary University of London, North-East London and Essex Regional Motor Neuron Disease Care Centre, London, E1 2AT, UK; 4 Faculty of Medicine, University of Southampton, MP801 University Hospital Southampton NHS Foundation Trust, SO16 6YD, UK; 5 Sheffield Institute for Translational Neuroscience (SiTraN), University of Sheffield, 385a Glossop Road, Sheffield, S10 2HQ, UK; 6 Department of Clinical Neuroscience, UCL Institute of Neurology, Rowland Hill Street, London, NW3 2PF, UK; 7 Department of Neurodegenerative Diseases, UCL Institute of Neurology, Queen Square, London WC1N 3BG, UK

**Keywords:** amyotrophic lateral sclerosis, neurodegeneration, complex trait, polygenic inheritance, association study

## Abstract

Amyotrophic lateral sclerosis is a progressive neurodegenerative disease of motor neurons. About 25 genes have been verified as relevant to the disease process, with rare and common variation implicated. We used next generation sequencing and repeat sizing to comprehensively assay genetic variation in a panel of known amyotrophic lateral sclerosis genes in 1126 patient samples and 613 controls. About 10% of patients were predicted to carry a pathological expansion of the *C9orf72* gene. We found an increased burden of rare variants in patients within the untranslated regions of known disease-causing genes, driven by *SOD1*, *TARDBP*, *FUS*, *VCP*, *OPTN* and *UBQLN2.* We found 11 patients (1%) carried more than one pathogenic variant (*P* = 0.001) consistent with an oligogenic basis of amyotrophic lateral sclerosis. These findings show that the genetic architecture of amyotrophic lateral sclerosis is complex and that variation in the regulatory regions of associated genes may be important in disease pathogenesis.

## Teaser

The genetic landscape of amyotrophic lateral sclerosis (ALS) is poorly understood. By examining known ALS-associated genes in a large cohort, Morgan, Shatunov *et al*. report an increase in mutations within the untranslated prime regions of the genes and a greater than expected number of patients with multiple potentially pathogenic variants.

## Introduction

Amyotrophic lateral sclerosis (ALS) is a fatal, neurodegenerative disorder with a life expectancy of 3–5 years from symptom onset. Presentation is with weakness of voluntary muscles, representing degeneration of the upper and lower motor neurons. The incidence of ALS is approximately 2 per 100 000 person–years with a slightly higher proportion of males (Logroscino *et al.*, 2010). About 15% have concomitant frontotemporal dementia (FTD) and up to 50% may have subtle cognitive impairment (Abrahams *et al.*, 2014).

The complexity of the molecular mechanisms implicated in ALS is paralleled by multifaceted genetics that are still not fully understood despite extensive research (Al-Chalabi and Hardiman, 2013; Morgan and Orrell, 2016). To address this issue, there has been a move towards next-generation sequencing (NGS) as a high-throughput, relatively inexpensive tool to uncover the genetic architecture of ALS and other heterogeneous neurological diseases, including Charcot–Marie–Tooth disease, dementia, ataxia and Parkinson’s disease (Johnson *et al.*, 2010; Mencacci *et al.*, 2014; Cady *et al.*, 2015; Cirulli *et al.*, 2015; van Rheenen *et al.*, 2016).

About 5–10% of ALS is considered familial (familial ALS), which may be an underestimate depending on the definition used (Byrne *et al.*, 2012), and there is extensive evidence that the distinction between familial and sporadic ALS is not clear-cut (Al-Chalabi and Lewis, 2011). Over 100 genes have been implicated in ALS to varying degrees (Abel *et al.*, 2012; http://alsod.iop.kcl.ac.uk), with ∼25 of these having been replicated in subsequent studies. The four most important ALS genes by frequency are *SOD1*, *TARDBP*/TDP-43, *C9orf72* and *FUS.* Variants in these genes are more likely to be of large effect, and carrying the genotype greatly increases the probability of ALS; in other words, these variants show moderate to high penetrance. Gene variants of low penetrance are also of interest, even though they only increase risk a little for any individual, as the overall variance in genetic risk explained is high, and such genes contribute to our understanding of the pathway to ALS. In at least some cases, ALS is oligogenic, with affected individuals carrying more than one rare variant implicated in ALS (van Blitterswijk *et al.*, 2012; Cady *et al.*, 2015). If a significant number of cases in ALS are indeed caused by more than one risk variant, this has implications for genetic counselling and treatment.

We therefore aimed to explore the genetics of ALS in a cohort of 1126 patients using a specific ALS-gene panel for NGS.

## Materials and methods

### Patients

A total of 1126 cases and 613 controls of European ancestry were used as part of this study. This was composed of 131 individuals with familial ALS (64 female, 67 male) and 995 with sporadic ALS (428 female, 567 male). The average age of onset was 56 years for familial ALS (range 24–85) and 61 for sporadic ALS (range 25–88). Control samples were composed of 232 females and 381 males. These clinical data are presented in Supplementary Table 1. Averaged across loci, 13 cases and 15 controls failed to sequence across some of the target genome. Three hundred and seventeen patients did not have complete *C9orf72* data because of insufficient DNA. Patient samples were obtained predominantly from the UK National DNA Bank for Motor Neuron Disease (MND) Research (Smith *et al.*, 2015) as well as from University College London and Partners (UCLP) MND clinics. A small subset (*n* = 95) of these samples overlap with our previous proof-of-principle publication and were included in this study to increase the power to detect mutation burden (Morgan *et al.*, 2015). Control samples were either sequenced using MiSeq technology (see below), whole-exome sequencing or both. They were selected based on their ethnicity, age (over 60 years old) and whether they were free from any neurological disorder. Additionally, subjects were excluded from the study if they had a first-degree relative with a neurological disorder including Alzheimer’s disease, ALS, ataxia, autism, bipolar disorder, cerebrovascular disease, dementia, dystonia, Parkinson’s disease and schizophrenia.

### Next-generation sequencing

An ALS-specific gene panel was designed for use on the Illumina MiSeq platform by means of the Illumina TruSeq Custom Amplicon Assay. This uses PCR amplicon-based target enrichment, and screens for variants across 23 genes that were selected based on their association with ALS, so that all the chief causal genes were included as well as several risk factors and a selection of variants with an uncertain relationship to ALS, either through a lack of evidence or through the gene more commonly causing a related disease. The panel included exons and flanking regions for *ALS2*, *ANG*, *CHMP2B*, *DAO*, *DCTN1*, *FIG4*, *FUS*, *NEFH*, *OPTN*, *PFN1*, *PON1*, *PON2*, *PON3*, *PRPH*, *SETX*, *SOD1*, *SQSTM1*, *TARDBP*, *TREM2*, *UBQLN2*, *VAPB*, *VCP* and *VEGFA.* The following genes were also extensively covered in both 5’ and 3’ untranslated regions (UTRs): three were selected because they are regarded as major ALS genes (*SOD1*, *TARDBP*, *FUS*) and three because the design of the assay made sequencing through the untranslated region simple to achieve (*OPTN*, *VCP*, *UBQLN2*; Supplementary Table 2). This study was initiated before the discovery of the ALS genes *C21orf2*, *CHCHD10*, *MATR3*, *NEK1*, *TBK1* and *TUBA4A.*

### Bioinformatic analysis

The raw FASTQ files from the Illumina MiSeq were aligned to the human reference genome build 19 (GRCh37) using Novoalign v3 hg19 (for transcripts see Supplementary Table 3), and variants were called using SAMtools v0.1.18 and GATK v3.3. Low quality variants were filtered as described previously (Morgan *et al.*, 2015). Annotation was performed using ANNOVAR Nov2014 (Wang *et al.*, 2010) and Variant Effect Predictor (VEP v84) tools (McLaren *et al.*, 2010) and compared against the ExAC database of genetic variants (http://exac.broadinstitute.org/), 1000 Genomes (www.1000genomes.org), ESP6500 (evs.gs.washington.edu), UK10K (http://www.uk10k.org/) and cg69 (www.completegenomics.com/public-data/69-Genomes) to remove common variants [minor allele frequency (MAF) > 1% of the European-derived population]. Any locus with significant missingness between cases and controls was excluded using PLINK v1.09 (Purcell *et al.*, 2007). An in-house coverage analysis software (CovCheck) determined that 92% of the desired regions were covered by at least 10 reads. The data were independently analysed by two separate groups to ensure different methods produced matching results, and every locus was visually inspected to guarantee high-quality data. Variants called with 10–20 reads were flagged to be visually inspected to remove false positives. A selection of these were checked using Sanger sequencing to ensure calling was correct.

### Repeat expansions

A major drawback of NGS is its inability to reliably assay variation represented by repeats such as those in *C9orf72* and *ATXN2.* Therefore, repeat-primed PCR was used to detect the expansion mutation in *C9orf72* and standard fragment length analysis for the microsatellite repeat in *ATXN2.* The primers and methods used for *C9orf72* and *ATXN2* repeat detection have been previously described (Pulst *et al.*, 1996; DeJesus-Hernandez *et al.*, 2011).

### Statistical analysis

**Figure 1:**
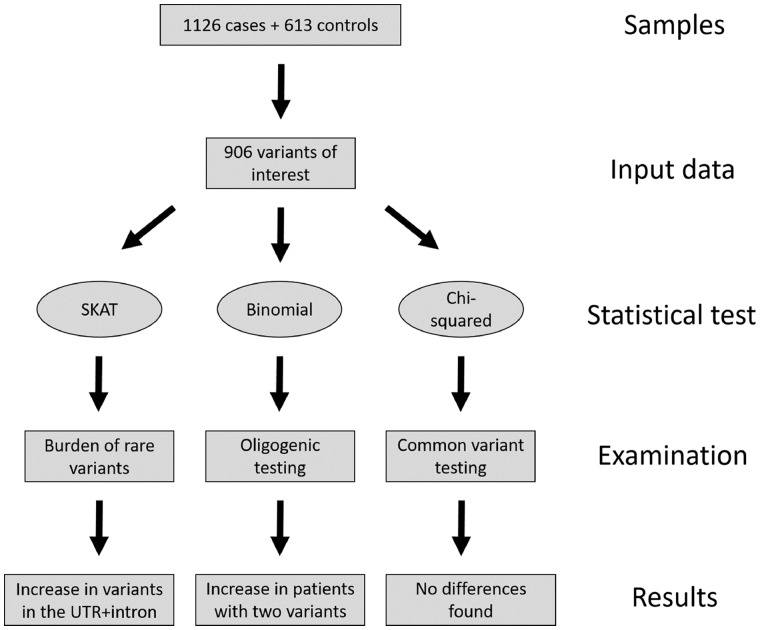
**Simplified flowchart describing the steps taken from sample to result in this study**.

Because rare variation is too infrequent to test statistically in a sample of this size, we used a region-based test comparing the rare-variant burden in cases and controls in the form of the SNP (single nucleotide polymorphism)-set sequence kernel association test (SKAT v1.1.2; Wu *et al.*, 2011; Fig. 1). We included intronic, exonic, synonymous, coding, and known causal variants only if they were novel or with a MAF < 0.01 and were of high-quality with no bias in the proportion of missing data between cases and controls. Comparing controls post-filtering, which had been examined using whole-exome and MiSeq-targeted sequencing, revealed differences only in the calling of indels of two or more nucleotides and not in any of the SNPs. We therefore used whole-exome sequenced samples as controls for SNP data only and did not include indels of two or more nucleotides in the analysis. The genes *C9orf72*, *ATXN2*, *PON1–3* and *VEGFA* were not included in SKAT analysis due to the nature of their association with the disease (repeat expansions or common variation), but were instead analysed separately as described below. Sex was used as a covariate. A total of five tests were carried out; adjusted *P*-values are reported using the formula:

(1) $$B=1-{\left(1-P\right)}^{n}$$

Where *P* is the critical *P*-value obtained in the test, *n* is the number of tests completed and *B* is the Bonferroni corrected *P*-value.

To examine a potential oligogenic basis of ALS, variants were analysed using a binomial test in R v3.2.3 as described previously (van Blitterswijk *et al.*, 2012; Fig. 1). Heterozygous and homozygous variants were treated as a single event and tested using the binomial distribution by the formula:

(2) $$f\left(x\right)=\left(\begin{array}{c} n\\ x \end{array}\right)p^{x}{\left(1-p\right)}^{\left(n-x\right)}\;wherex=0,1,2,\ldots ,n$$

Where *f*(*x*) is the probability of observing more than one rare variant in a single individual, *n* is the total number of patients, *x* the number of people carrying more than one variant, and *p* is the expected frequency of rare variation derived from the averaged frequency of rare variation observed in cases and controls.

We performed this test twice, first on reported ALS-variants only and then on variation found in *C9orf72*, *SOD1*, *TARDBP*, *FUS*, *ANG*, *ALS2*, *VCP*, *OPTN*, *NEFH* and *UBQLN2* where we included all rare (MAF < 0.01), exonic, coding variation in these genes (excluding *C9orf72* where only repeat expansions were included). These 10 genes were selected based on those previously tested by van Blitterswijk *et al.* (2012) in addition to those we believed to be of importance to the disease. Reported *P*-values were corrected for two tests.

For variants in the genes *PON1–3* and *VEGFA*, association with ALS is with common variation, and so these loci were selected out of the data and filtered for variants present in dbSNP (Sherry *et al.*, 2001; Fig. 1). Chi-squared SNP-based association tests in cases versus controls were performed using PLINK for these 20 loci and corrected for each iteration.

## Results

We performed NGS on 1126 cases and 613 controls that identified, on average, 31 variants per individual passing quality control filters. Findings are presented in Table 1.

**Table 1:** Previously published variants in ALS or other diseases that have been found in the current study in either patients or controls

Chromo- some	Base pair	Gene	1000 g	UK10K	ExAC	dbSNP137	PhyloP	SIFT	PolyPhen2	LRT	Exon	Nucleotide change	Amino acid change	Patients, *n*	Controls, *n*
1	11076931	*TARDBP*	.	0.00052	2.11 × 10^−4^	rs80356715	C	B	B	D	3	C269T	A90V	2	1
1	11082325	*TARDBP*	.	.	8.13 × 10^−6^	rs80356719	C	B	B	D	6	G859A	G287S	2	0
1	11082428	*TARDBP*	.	.	.	.	C	D	B	D	6	C962T	A321V	1	0
1	11082475	*TARDBP*	.	.	.	rs80356730	C	B	D	D	6	A1009G	M337V	1	0
1	11082509	*TARDBP*	.	.	.	.	C	D	P	D	6	G1043T	G348V	1	0
1	11082588	*TARDBP*	.	.	.	.	N	NA	NA	D	6	T1122G	Y374X	1	0
1	11082598	*TARDBP*	.	.	.	.	C	D	B	D	6	A1132G	N378D	2	0
2	74588717	*DCTN1*	0.0005	0.0047	3.11 × 10^−3^	rs72466496	C	D	B	N	32	C3746T	T1249I	0	5
2	74594023	*DCTN1*	.	0.00078	1.95 × 10^−4^	rs121909344	C	D	D	N	21	C2353T	R785W	1	1
2	202626437	*ALS2*	0.02	0.026	0.025	rs3219154	N	B	B	N	4	A280G	I94V	0	33
3	87289899	*CHMP2B*	.	0.00052	1.22 × 10^−4^	rs63750818	N	B	B	N	2	A85G	I29V	2	2
3	87294943	*CHMP2B*	.	0.00026	4.07 × 10^−5^	rs200792883	C	D	D	D	3	G206A	R69Q	1	0
3	87294985	*CHMP2B*	.	.	.	.	C	B	D	D	3	C248T	T83I	0	1
5	179251013	*SQSTM1*	.	.	2.20 × 10^−4^	rs145056421	N	B	B	N	3	G457A	V153I	0	1
5	179252184	*SQSTM1*	0.0032	0.0018	2.42 × 10^−3^	rs11548633	C	B	D	D	5	A712G	K238E	11	4
5	179263445	*SQSTM1*	0.0046	0.0021	8.78 × 10^−4^	rs104893941	C	D	P	N	8	C1175T	P392L	5	2
6	41129105	*TREM2*	0.03	0.0023	0.012	rs2234253	C	D	D	D	2	C287A	T96K	0	1
6	41129207	*TREM2*	0.01	0.0088	8.25 × 10^−3^	rs143332484	N	B	B	N	2	G185A	R62H	0	10
6	41129252	*TREM2*	0.0027	0.0021	2.07 × 10^−3^	rs75932628	C	B	D	N	2	G140A	R47H	7	4
6	110036336	*FIG4*	0.0009	0.0031	9.68 × 10^−4^	rs121908287	C	D	D	D	2	T122C	I41T	3	5
9	35066777	*VCP*	.	.	8.95 × 10^−5^	.	C	B	B	D	4	A340G	I114V	1	0
9	35068298	*VCP*	0.0009	0.00052	5.37 × 10^−4^	rs140913250	C	B	B	D	2	A79G	I27V	0	1
9	135140020	*SETX*	0.0027	0.0078	3.40 × 10^−3^	rs151117904	N	D	B	N	26	T7640C	I2547T	12	8
9	135204004	*SETX*	0.0018	.	5.77 × 10^−4^	rs149546633	N	D	B	N	10	A2981G	D994G	0	1
9	135204010	*SETX*	0.01	0.013	0.015	rs61742937	N	D	B	N	10	A2975G	K992R	0	16
9	135224757	*SETX*	0.01	0.0075	9.04 × 10^−3^	rs79740039	N	D	B	N	3	G59A	R20H	0	13
10	13152400	*OPTN*	0.07	0.033	0.045	rs11258194	N	B	B	N	5	T293A	M98K	55	33
10	13178802	*OPTN*	.	.	.	.	C	D	D	N	16	A1670G	K557R	0	1
12	49689009	*PRPH*	0.02	0.0068	0.014	rs57451017	C	D	P	N	1	G26A	R9Q	0	12
12	49690798	*PRPH*	0.03	0.016	0.022	rs62636520	C	B	B	N	4	G829A	A277T	0	20
14	21161845	*ANG*	0.0005	0.0031	1.42 × 10^−3^	rs121909536	N	D	B	U	2	A122T	K41I	0	2
14	21161973	*ANG*	0.01	.	1.55 × 10^−3^	rs17560	N	B	B	U	2	A250G	K84E	1	0
16	31201719	*FUS*	0.0009	.	6.51 × 10^−5^	rs186547381	C	D	D	D	12	C1292T	P431L	1	0
16	31202410	*FUS*	.	.	.	.	C	D	D	D	14	G1520A	G507D	1	0
16	31202739	*FUS*	.	.	.	rs121909670	N	D	D	D	15	C1561T	R521C	1	0
16	31202740	*FUS*	.	.	.	rs121909671	N	D	D	D	15	G1562A	R521H	2	0
16	31202740	*FUS*	.	.	.	.	N	D	D	D	15	G1562T	R521L	1	0
17	4849268	*PFN1*	0.0005	.	4.15 × 10^−4^	rs140547520	C	D	D	D	3	A350G	E117G	1	1
20	57014075	*VAPB*	0.0009	0.0026	1.34 × 10^−3^	rs146459055	C	B	B	D	4	T390G	D130E	0	1
20	57016076	*VAPB*	0.0018	0.0013	1.35 × 10^−3^	rs143144050	C	B	P	D	5	G510A	M170I	7	1
20	57016117	*VAPB*	.	0.0005	.	rs145483046	C	B	B	N	5	G551A	R184Q	1	0
21	33032107	*SOD1*	.	.	.	.	N	D	B	N	1	C25G	L9V	1	0
21	33038821	*SOD1*	.	.	.	.	C	NA	D	D	3	G229T	D77Y	3	0
21	33039603	*SOD1*	0.0009	0.00078	1.11 × 10^−3^	rs80265967	N	B	B	N	4	A272C	D91A	3	0
21	33039636	*SOD1*	.	.	.	.	C	NA	D	D	4	A305G	D102G	1	0
21	33039650	*SOD1*	.	.	.	.	C	NA	D	D	4	C319T	L107F	1	0
21	33039666	*SOD1*	.	.	.	.	N	NA	NA	D	4	G335A	C112Y	1	0
21	33039672	*SOD1*	.	0.00026	.	rs121912441	C	NA	D	D	4	T341C	I114T	5	0
21	33040829	*SOD1*	.	.	.	.	C	NA	NA	D	5	A403G	S135G	1	0
22	29885016	*NEFH*	0.04	0.09	0.069	rs59371099	C	B	NA	D	4	G1387A	E463K	173	109
22	29885473	*NEFH*	0.17	0.23	0.182	rs5763269	C	D	D	D	4	C1844T	P615L	414	205
22	29885997	*NEFH*	.	.	8.78 × 10^−4^	rs59551486	.	.	.	.	4	2368_2370 delAAG	K790del	1	2
22	29886043	*NEFH*	0.12	0.17	0.153	rs165602	C	D	NA	N	4	A2414C	E805A	309	152
X	56591796	*UBQLN2*	.	.	.	.	C	B	NA	N	1	C1490A	P497H	1	0

The references for these variants are found in Supplementary Table 3.

1000g = 1000 Genomes project; prediction scores: C = conserved; D = damaging; B = benign; N = not conserved; NA = not applicable.

### Variant interpretation

One of the major difficulties in NGS data is how to interpret the pathogenicity of variants, especially those that are novel or extremely rare. We found 906 alterations that were defined as potentially pathogenic, of which 225 were exonic and 55 were previously published as associated with ALS (48 variants) or another disease. However, some of these variants were also found in the control cohort (Table 1). We therefore defined variants as potentially pathogenic for ALS if they were published previously in more than one study and not found in control cohorts. Under this interpretation, 109 of 1125 cases were predicted to carry a pathological expansion of the *C9orf72* gene, 19 cases with a likely causal *SOD1* mutation, 14 within *TARDBP* and nine with a *FUS* variant.

### Burden of rare variants

SKAT analysis showed an increased number of rare variants in cases compared to controls (*P* = 0.003). As this may be solely due to known pathogenic variants, those previously reported in ALS were excluded, regardless of putative pathogenicity, and SKAT was repeated. The result was still significant (*P* = 0.01). The burden lay primarily in the UTR and intronic regions rather than exons. We therefore tested introns and UTRs (*P* = 0.04) independently of exons [*P* = 0.1 (synonymous) and *P* = 0.1 (non-synonymous)]. Additionally, there were more rare variants in UTRs than in introns in patients but we did not have the statistical power to investigate this in detail (see Supplementary Table 5 for full results).

### Oligogenic analysis

A binomial test restricted to exonic variants previously published in ALS did not show an excess of patients with two mutations (*P* = 0.4; see Supplementary Table 4 for variant references). Unrestricted testing of exons and adjacent regions for *C9orf72*, *SOD1*, *TARDBP*, *FUS*, *ANG*, *ALS2*, *VCP*, *OPTN*, *NEFH* and *UBQLN2* showed 11 patients with more than one mutation, significantly higher than expected by chance based on the mutation rates in cases and controls (*P* = 0.001; see Supplementary Table 6 for variant and patient information).

### Common variation in amyotrophic lateral sclerosis

The genes *PON1–3* and *VEGFA* have both been reported as potential risk factors for ALS. We selected 20 loci of common variation within these genes to analyse but did not find any significant differences in SNP frequencies between controls and cases and, in fact, some reported important SNPs were present at a higher rate in controls than in cases (Supplementary Table 7). The frequencies in our cohort were higher than those observed in the ExAC database.

## Discussion

We analysed 24 ALS genes in 1736 subjects. We detected 55 variants previously published in ALS or other diseases and 845 rare variants of uncertain significance, with a higher burden of variants in cases, an excess in the untranslated regions and introns, and oligogenic inheritance in 1% of patients. A limitation of this study is its focus on a specific set of ALS genes rather than being a truly unbiased survey of the exome or genome, but this has allowed us to test specific hypotheses. Cases with likely pathogenic *SOD1*, *TARDBP* and *FUS* mutations are more likely to report a family history while those with a *C9orf72* expansion are more commonly sporadic and bulbar onset.

### Untranslated region analysis in amyotrophic lateral sclerosis

The UTRs of genes are often ignored in genetic studies of disease, partly because of the difficulty in interpreting findings. We included UTRs to address this lack of knowledge targeting *SOD1*, *TARDBP*, *FUS*, *OPTN*, *VCP* and *UBQLN2*, as well as partial coverage in the remaining genes. SKAT analysis revealed a significant excess of rare variants in patients in the UTRs and introns, and inspection of the data suggested that the UTRs contained most of this burden. Previous studies have also suggested this effect (Fig. 1). An Italian study of 420 people with ALS and 480 controls found non-coding mutations in the 3’UTR of *FUS* in patients, with four unique rare variants in five individuals and no rare variants in controls (Sabatelli *et al.*, 2013). Three variants were studied further in primary fibroblast cultures (c.^*^59G > A, c.^*^108C > T and c.^*^110G > A). The UTR variants and a known pathogenic exonic *FUS* variant all cause a mislocalization of the FUS protein, an effect not seen in the other patients or controls. Similarly, the c.^*^48G > A variant, found in two subjects with a rapidly progressive form of ALS, increase FUS expression dramatically (Dini Modigliani *et al.*, 2014); overexpression of wild-type FUS causes an ALS-like syndrome in mice (Mitchell *et al.*, 2013). The 3’UTR of *FUS* is known to be involved in a feedback loop for its own expression via the alternative splicing of exon 7 (Zhou *et al.*, 2013). On the other hand, FUS-knockout mice exhibit abnormalities but not ALS (Kino *et al.*, 2015), and the ExAC database shows no loss of function *FUS* variants in any of the 58 787 individuals sequenced despite a statistical expectation of there being 28.6. This number is calculated using the mutation rate across the whole genome to produce a per gene probability of each type of mutation. Factoring in coverage metrics, an expected number of variants, in this case 28.6, can be obtained for the number of individuals sequenced (Lek *et al.*, 2015). These studies combined suggest that tight control of FUS expression is necessary in humans (Dini Modigliani *et al.*, 2014).

There are similar findings for *TARDBP*: a 3’ UTR variant c.^*^2076G > A found in two affected members of a family with ALS and FTD (Gitcho *et al.*, 2009), doubled *TARDBP* RNA expression and was not present in 982 controls. Like *FUS*, *TARDBP* also regulates its own expression through the 3’UTR (Ayala *et al.*, 2011), although this is achieved through RNA instability rather than splicing. Again, as with *FUS*, there are no loss-of-function variants in *TARDBP* in the ExAC database when 11.8 are expected.

**Figure 2:**
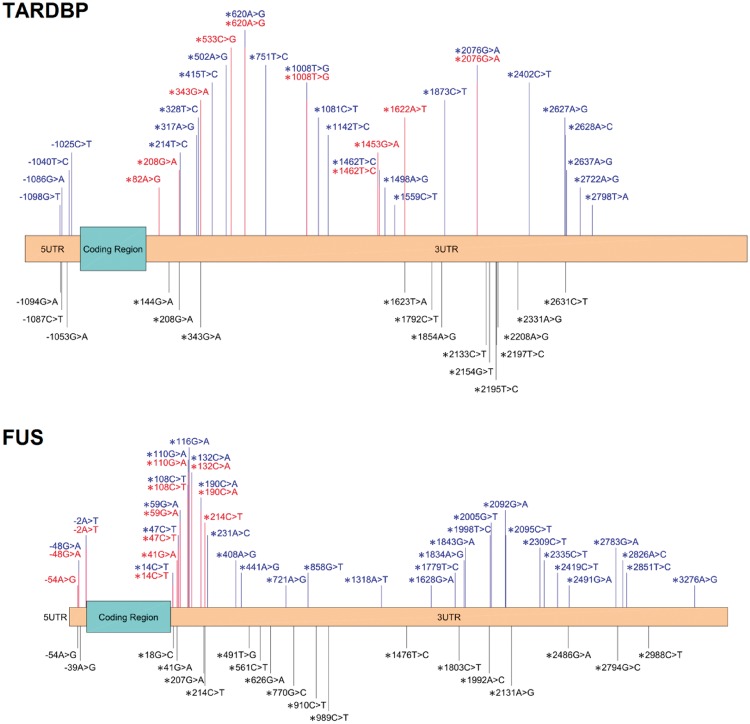
**Schematic representation of the UTRs of *TARDBP* and *FUS* and the variants found in this study within cases (blue) and controls (black) in these regions.** Variants within the 5’UTR are preceded with a minus while those contained in the 3’UTR are headed by an asterisk. This includes variants previously published in ALS marked in red (Supplementary Table 8).

These findings suggest variants in the UTRs of *FUS* and *TARDBP* have a role in ALS pathogenesis (Fig. 2).

### Oligogenic basis of amyotrophic lateral sclerosis

We implemented a binominal test based on the probability distribution of mutations in both cases and controls to identify a greater than chance observation of combined pathogenic mutations. There was no excess of oligogenic ALS when the analysis was restricted to previously reported ALS variants, but previous studies have been unrestricted, examining all rare variants in targeted genes (van Blitterswijk *et al.*, 2012). Performing this analysis, 1% of our patients had two mutations, significantly higher than expected based on known mutation rates. Most had *C9orf72* repeat expansion combined with another mutation (e.g. *VCP* R155H or *TARDBP* A321V; Supplementary Table 6). A single control also had two mutations, P372R in *ALS2* and A90V in *TARDBP. ALS2* pathogenicity has only been observed in homozygotes, and this individual was heterozygous. Furthermore, the *TARDBP* variant has been previously identified in controls and has unclear status, although it is associated with abnormal localization and aggregation of *TARDBP* (Guerreiro *et al.*, 2008; Winton *et al.*, 2008).

What constitutes a pathogenic combination of mutations is debatable as some variants are of uncertain significance (Richards *et al.*, 2015), and the combination of a pathogenic variant with one of uncertain significance has been considered oligogenic inheritance by some (Nakamura *et al.*, 2016). Similarly, variation in *ANG* or *NEFH* is generally considered a weak contributor to ALS risk, and missense variants in *SPG11* are often benign unless resulting in loss of function. We find oligogenic inheritance even when these genes are excluded from the analysis. Notably, one of our controls harboured a loss-of-function mutation in *SPG11.* Allowing a looser definition of oligogenic inheritance, oligogenic ALS is reported in ∼1.6% of cases (4% in familial and 1.3% in sporadic ALS) (Kenna *et al.*, 2013). Oligogenic ALS involving the convergence of two ALS families has also been observed, with the proband carrying a pathogenic *TARDBP* variant and *C9orf72* repeat expansion (Chiò *et al.*, 2012). This appears to be associated with a more severe phenotype and an earlier age of onset. In another study of 391 cases, 3.8% had more than one mutation and an earlier age of onset by 10 years (Cady *et al.*, 2015).

### Common variation

Several previous studies have examined common variations in *VEGFA* and *PON1–3*, which are inconsistently associated with ALS (Lambrechts *et al.*, 2003; Wills *et al.*, 2009). We characterized 20 loci in patients and controls to find no relationship with ALS, but this may be due to low call rates in patients for these particular genes (Supplementary Table 7). For example, rs7493 and rs12026 were associated with controls, but only 95 cases had adequate data. Furthermore, 14 of the 20 tested loci were at higher frequencies in our controls than in the ExAC and 1000 Genomes databases, with six being considerably higher. This highlights the importance of collecting adequate controls for each study rather than solely relying on public data.

The sole known pathogenic *DAO* variant R199W has been reported in two studies and correlates with survival (Mitchell *et al.*, 2010; Cirulli *et al.*, 2015). We identified two subjects with sporadic ALS who harboured a different variant at the same codon: R199Q, and another alteration two amino acids away: Q201R. Both of these *DAO* mutants are predicted to be damaging by PhyloP, SIFT, PolyPhen and LRT, and were not found in our control samples.

Our large-scale sequencing study in ALS has identified a number of rare variations, many novel, and shown that the UTR of *TARDBP* and *FUS* are potentially important in the pathogenesis of ALS. We have also provided further support for oligogenic inheritance of ALS in a proportion of cases.

## Supplementary Material

Supplementary Data

## Abbreviations

ALS: amyotrophic lateral sclerosis
MND: motor neuron disease
NGS: next-generation sequencing
SKAT: sequence kernel association test
SNP: single nucleotide polymorphism
UTR: untranslated region
