# Supplementary material for: A comprehensive analysis of rare genetic variation in amyotrophic lateral sclerosis in the UK
Source: Brain. 2017 Apr 18;140(6):1611–8. doi: 10.1093/brain/awx082 (PMC5445258; doi:10.1093/brain/awx082)
Supplement: Supplementary Data [file awx082_Supp.pdf]

Supplementary material

Variation in untranslated regions

There are a number of studies which report UTR variants in ALS with uncertain pathogenicity. Rutherford *et al.* (2008) report six UTR alterations in *TARDBP* but do not reveal how many cases each variant was in or if these were present in controls. In 2009, a French cohort of 285 sporadic ALS cases were sequenced to reveal one patient harbouring c.*1462T>C in *TARDBP* which was not in 360 controls (Daoud *et al.*, 2009). Then four UTR alterations were reported in this gene in 410 ALS/FTD cases in Belgium (Gijselinck *et al.*, 2009). Two *FUS* 3'UTR changes were present in an Italian ALS cohort but the authors did not reveal if they were present in their 376 controls which seems likely given that c.*41G>A is a common polymorphism (Ticozzi *et al.*, 2009). The *FUS* c.*24G<C change was detected in a fALS proband which was absent from 970 controls and two affected relatives indicating it is likely to be a rare polymorphism (Groen *et al.*, 2010). Another study revealed UTR variants in *FUS* and *SOD1* in ALS but it is not explicitly stated if they also sequenced their 700 controls for these regions (DeJesus-Hernandez *et al.*, 2010). Drepper *et al.* (2011) and Zou *et al.* (2012) both report these non-coding *FUS* changes in ALS that are absent from controls while in *VCP*, c.*12C<T is present in cases and not 1,205 controls (Abramzon *et al.*, 2012). Lastly, *ANG* and *FUS* were reported to have UTR mutations not present in controls, one of which we also found solely in cases, namely c.*132C<A (Brown *et al.*, 2012). While this collection of papers seem to indicate a burden of UTR variation in ALS, not a single report mentions if any rare variation existed solely in controls. This piece of information is lacking in many publications and not just for UTR data.

Oligogenic ALS

van Blitterswijk *et al.* (2012a) found five families with multiple mutations (5% of their familial cohort) which was statistically more than that expected by chance. Another study by van Blitterswijk *et al.* (2012b) found a novel *VAPB* variant alongside the *C9orf72* mutation, however, the pathogenicity of the *VAPB* mutation was not confirmed. Lastly, Bury *et al.* (2016) published a patient with mutations in both *OPTN* and *C9orf72*. The aggregates within motor and non-motor neuronal cells were studied in this patient to reveal OPTN staining in aggregates even in cells absent for TARDBP-positive inclusions.

Common variation

OPTN is known to cause both ALS and primary open angle glaucoma (POAG) although individual mutations appear to only cause one of these diseases and never both, suggesting that there are completely different mechanisms by which it causes each (Swarup *et al.,* 2013). We identified two known variants which are both reported to cause POAG: N303K and R545Q (Rezaie *et al.*, 2002; Mukhopadhyay *et al.*, 2005; Buentello-Volante *et al.*, 2013). The former is present in a sporadic ALS patient and the latter in both one control and one person with sporadic ALS, suggesting that these variants do not cause POAG or ALS. These findings challenge the current literature and imply a difficulty in characterising novel variants found in a disease, particularly if insufficient controls have been sequenced.

Other variants previously reported which have been found in our controls include the *CHMP2B* I29V (0.2% of cases and 0.3% of controls). This variant was found in ALS patients (Parkinson *et al.*, 2006) while functional work in cells indicated that it causes cytoplasmic vacuoles (Cox *et al.*, 2010). The K238E variant in *SQSTM1* was found in two studies restricted to ALS (Rubino *et al.*, 2012; Cady *et al.*, 2015) but only the former sequenced controls (n=145) whereas four of our control group had this mutation (0.7%). A single control patient had the I27V alteration in *VCP* which has been described in FTD and not in

461 controls (Rohrer *et al.*, 2011; Beck *et al.*, 2014), nevertheless, the latter group identified I27V in combination with another mutation and so some of these variants may be risk factors for disease rather than causal alone.

Supplementary Table 1. Summary of patients used in this study.

	Total number	Female	Male	Average age of onset (range)	ALS-Definite	ALS-Probable	PMA/PLS/PBP
Familial	131	49%	51%	56 (24-85)	44%	49%	7%
Sporadic	995	43%	57%	61 (25-88)	38%	49%	13%
Control	613	38%	62%	N/A	N/A	N/A	N/A

Supplementary Table 2. Overview of coverage for each gene within coding and non-coding regions.

Gene	Exon	Intron	UTR	Average coverage
ALS2	Part	Part	None	103x
ANG	Full	Part	Full	139x
CHMP2B	Full	Part	Part	104x
DAO	Part	Part	None	208x
DCTN1	Part	Part	None	132x
FIG4	Part	Part	None	139x
FUS	Full	Part	Full	165x
NEFH	Part	None	None	150x
OPTN	Full	Part	Full	112x
PFN1	Full	Part	Part	86x
PON1	Part	Part	Part	115x
PON2	None	Part	None	42x
PON3	Part	Part	None	68x
PRPH	Part	Part	None	55x
SETX	Part	Part	None	121x
SOD1	Full	Part	Full	154x
SQSTM1	Part	Part	Part	81x
TARDBP	Full	Part	Full	232x
TREM2	Part	Part	None	132x
UBQLN2	Full	N/A	Full	122x
VAPB	Part	Part	None	150x
VCP	Full	Part	Full	180x
VEGFA	Part	Part	Part	30x

Supplementary Table 3. List of transcripts used in this study.

Gene	Transcript
ALS2	NM_020919
ANG	NM_001145
CHMP2B	NM_014043
DAO	NM_001917
DCTN1	NM_004082
FIG4	NM_014845
FUS	NM_004960
NEFH	NM_021076
OPTN	NM_001008211
PFN1	NM_005022
PON1	NM_000446
PON2	NM_001018161
PON3	NM_000940
PRPH	NM_006262
SETX	NM_015046
SOD1	NM_000454
SQSTM1	NM_003900
TARDBP	NM_007375

TREM2	NM_018965
UBQLN2	NM_013444
VAPB	NM_004738
VCP	NM_007126
VEGFA	NM_001025366

Supplementary Table 4. Extension of Table 1 of the list of previously reported variants and the references associated with them. SP = spastic paraplegia; CMT = Charcot-Marie-Tooth disease; PD = Parkinson's disease; ET = essential tremor; POAG = primary open angle glaucoma; NS = not significant;

Chromosome	Base pair	Gene	Nucleotide change	Amino acid change	No. patients	No. controls	Reference(s)
1	11076931	TARDBP	C269T	A90V	2	1	Guerreiro *et al.*, 2008; Winton *et al.*, 2008
1	11082325	TARDBP	G859A	G287S	2	0	Kabashi *et al.*, 2008
1	11082428	TARDBP	C962T	A321V	1	0	Kirby *et al.*, 2010
1	11082475	TARDBP	A1009G	M337V	1	0	Sreedharan *et al.*, 2008
1	11082509	TARDBP	G1043T	G348V	1	0	Kirby *et al.*, 2010
1	11082588	TARDBP	T1122G	Y374X	1	0	Daoud *et al.*, 2009
1	11082598	TARDBP	A1132G	N378D	2	0	Tsai *et al.*, 2011
2	74588717	DCTN1	C3746T	T1249I	0	5	Cady *et al.*, 2015; Münch *et al.*, 2004
2	74594023	DCTN1	C2353T	R785W	1	1	Münch *et al.*, 2004
2	202626437	ALS2	A280G	I94V	0	33	NS: Hand *et al.*, 2003; SP: Herzfeld *et al.*, 2009
3	87289899	CHMP2B	A85G	I29V	2	2	Cox *et al.*, 2010; Parkinson *et al.*, 2006
3	87294943	CHMP2B	G206A	R69Q	1	0	van Blitterswijk *et al.*, 2012c
3	87294985	CHMP2B	C248T	T83I	0	1	van Blitterswijk *et al.*, 2012c
5	179251013	SQSTM1	G457A	V153I	0	1	Cady *et al.*, 2015
5	179252184	SQSTM1	A712G	K238E	11	4	Cady *et al.*, 2015; Rubino *et al.*, 2012
5	179263445	SQSTM1	C1175T	P392L	5	2	Fecto *et al.*, 2011
6	41129105	TREM2	C287A	T96K	0	1	Dementia: Guerreiro *et al.*, 2013
6	41129207	TREM2	G185A	R62H	0	10	FTD: Lattante *et al.*, 2013
6	41129252	TREM2	G140A	R47H	7	4	Cady *et al.*, 2014; Rayaprolu *et al.*, 2013
6	110036336	FIG4	T122C	I41T	3	5	CMT: Chow *et al.*, 2007; Lenk *et al.*, 2011
9	35066777	VCP	A340G	I114V	1	0	CMT: Gonzalez *et al.*, 2014
9	35068298	VCP	A79G	I27V	0	1	FTD: Beck *et al.*, 2014; Rohrer *et al.*, 2011
9	135140020	SETX	T7640C	I2547T	12	8	Arning *et al.*, 2012; Rudnik-Schöneborn *et al.*, 2012
9	135204004	SETX	A2981G	D994G	0	1	Cady *et al.*, 2015
9	135204010	SETX	A2975G	K992R	0	16	Arning *et al.*, 2012; Nanetti *et al.*, 2013
9	135224757	SETX	G59A	R20H	0	13	Arning *et al.*, 2012
10	13152400	OPTN	T293A	M98K	55	33	POAG: Rezaie *et al.*, 2002
10	13178802	OPTN	A1670G	K557R	0	1	Del Bo *et al.*, 2011
12	49689009	PRPH	G26A	R9Q	0	12	Gros-Louis *et al.*, 2004
12	49690798	PRPH	G829A	A277T	0	20	Corrado *et al.*, 2011; Gros-Louis *et al.*, 2004
14	21161845	ANG	A122T	K41I	0	2	Cady *et al.*, 2015; Greenway *et al.*, 2006
14	21161973	ANG	A250G	K84E	1	0	Brown *et al.*, 2012; Cady *et al.*, 2015
16	31201719	FUS	C1292T	P431L	1	0	ET: Rajput *et al.*, 2014
16	31202410	FUS	G1520A	G507D	1	0	Corrado *et al.*, 2010
16	31202739	FUS	C1561T	R521C	1	0	Suzuki *et al.*, 2010; Tateishi *et al.*, 2010
16	31202740	FUS	G1562A	R521H	2	0	Blair *et al.*, 2010; FTD: van Langenhove *et al.*, 2010
16	31202740	FUS	G1562T	R521L	1	0	Deng *et al.*, 2010; Zou *et al.*, 2012
17	4849268	PFN1	A350G	E117G	1	1	Fratta *et al.*, 2014; Wu *et al.*, 2012
20	57014075	VAPB	T390G	D130E	0	1	Cady *et al.*, 2015
20	57016076	VAPB	G510A	M170I	7	1	van Blitterswijk *et al.*, 2012b
20	57016117	VAPB	G551A	R184Q	1	0	PD: Kun-Rodrigues *et al.*, 2015
21	33032107	SOD1	C25G	L9V	1	0	Andersen *et al.*, 2003
21	33038821	SOD1	G229T	D77Y	3	0	Cady *et al.*, 2015; Eisen *et al.*, 2008
21	33039603	SOD1	A272C	D91A	3	0	Robberecht *et al.*, 1996
21	33039636	SOD1	A305G	D102G	1	0	Ayers *et al.*, 2014; Orrell *et al.*, 1999
21	33039650	SOD1	C319T	L107F	1	0	Hineno *et al.*, 2012; Soong *et al.*, 2014
21	33039666	SOD1	G335A	C112Y	1	0	Eisen *et al.*, 2008; Nakamura *et al.*, 2012
21	33039672	SOD1	T341C	I114T	5	0	Kokubo Y *et al.*, 1999; Lopate *et al.*, 2010
21	33040829	SOD1	A403G	S135G	1	0	Conte *et al.*, 2012
22	29885016	NEFH	G1387A	E463K	173	109	Daoud *et al.*, 2011
22	29885473	NEFH	C1844T	P615L	414	205	Daoud *et al.*, 2011
22	29885997	NEFH	2368_2370delAAG	K790del	1	2	Figlewicz *et al.*, 1994
22	29886043	NEFH	A2414C	E805A	309	152	with concussion: Covassin, 2010
X	56591796	UBQLN2	C1490A	P497H	1	0	Deng *et al.*, 2011

Supplementary Table 5. List of non-exonic variants found in cases and controls.

Gene	Chromosome	Base pair	Location	Variant	1000G	ESP6500	CG69	ExAC	dbSNP137	Frequency Controls	Frequency Patients
TARDBP	1	11072687	UTR5	c.-1098G>T	.	.	.	.	.	0.16%	0.44%
TARDBP	1	11072698	UTR5	c.-1087C>T	.	.	.	.	.	0.49%	0%
TARDBP	1	11072699	UTR5	c.-1086G>A	.	.	.	.	.	0.16%	0%
TARDBP	1	11072732	UTR5	c.-1053G>A	.	.	.	.	.	0%	0.44%
TARDBP	1	11072745	UTR5	c.-1040T>C	.	.	.	.	.	0%	0.36%
TARDBP	1	11072760	UTR5	c.-1025C>T	.	.	.	.	.	0.33%	0%
TARDBP	1	11072802	splicing	T>C	.	.	.	.	.	0%	0.27%
TARDBP	1	11072810	intronic	G>A	.	.	.	.	.	0%	0.44%
TARDBP	1	11072831	intronic	C>G	.	.	.	.	.	0%	0.44%
TARDBP	1	11074031	intronic	C>T	.	.	.	1.04E-03	.	0%	0.44%
TARDBP	1	11076870	intronic	T>C	.	.	.	.	.	0%	0.27%
TARDBP	1	11076886	intronic	G>A	.	0.000692	.	2.68E-04	rs200066188	0%	0.44%
TARDBP	1	11077083	intronic	A>G	0.0005	.	.	1.63E-05	rs200818944	0%	0.44%
TARDBP	1	11082171	intronic	T>A	.	.	.	.	.	0%	0.36%

TARDBP	1	11082855	UTR3	c.*144G>A	.	.	.	.	.	0%	0.44%
TARDBP	1	11082925	UTR3	c.*214T>C	.	.	.	.	.	0%	0.44%
TARDBP	1	11083028	UTR3	c.*317A>G	.	.	.	.	.	0%	0.36%
TARDBP	1	11083039	UTR3	c.*328T>C	.	.	.	.	.	0.16%	0%
TARDBP	1	11083054	UTR3	c.*343G>A	.	.	.	.	.	0%	0.44%
TARDBP	1	11083126	UTR3	c.*415T>C	.	.	.	.	.	0%	0.44%
TARDBP	1	11083213	UTR3	c.*502A>G	.	.	.	3.19E-04	.	0%	0.44%
TARDBP	1	11083257	UTR3	c.*546delC	.	.	.	.	.	0.16%	0%
TARDBP	1	11083331	UTR3	c.*620A>G	.	.	.	1.35E-04	.	0.49%	0%
TARDBP	1	11083462	UTR3	c.*751T>C	.	.	.	.	.	0.33%	0%
TARDBP	1	11083719	UTR3	c.*1008T>G	0.0014	.	.	.	rs141412238	0.16%	0%
TARDBP	1	11083792	UTR3	c.*1081C>T	0.0009	.	0.007	.	rs184303021	0%	0.09%
TARDBP	1	11083853	UTR3	c.*1142T>C	.	.	.	.	.	0%	0.09%
TARDBP	1	11084209	UTR3	c.*1498A>G	.	.	.	.	.	0%	0.18%
TARDBP	1	11084270	UTR3	c.*1559C>T	.	.	.	.	.	0%	0.18%
TARDBP	1	11084503	UTR3	c.*1792C>T	.	.	.	.	.	0%	0.09%
TARDBP	1	11084565	UTR3	c.*1854A>G	0.0014	.	.	.	rs185335376	0%	0.09%
TARDBP	1	11084584	UTR3	c.*1873C>T	.	.	.	.	.	0%	0.09%
TARDBP	1	11084844	UTR3	c.*2133C>T	.	.	.	.	.	0%	0.27%
TARDBP	1	11084865	UTR3	c.*2154G>T	.	.	.	.	.	0%	0.09%
TARDBP	1	11084906	UTR3	c.*2195T>C	.	.	.	.	.	0%	0.09%
TARDBP	1	11084908	UTR3	c.*2197T>C	.	.	.	.	.	0%	0.09%
TARDBP	1	11084919	UTR3	c.*2208A>G	.	.	.	.	.	0%	0.27%
TARDBP	1	11085113	UTR3	c.*2402C>T	.	.	.	.	.	0.98%	0.09%
TARDBP	1	11085338	UTR3	c.*2627A>G	.	.	.	.	.	0.98%	0%
TARDBP	1	11085339	UTR3	c.*2628A>C	.	.	.	.	.	0.49%	0%
TARDBP	1	11085342	UTR3	c.*2631C>T	.	.	.	.	.	0%	0.09%
TARDBP	1	11085348	UTR3	c.*2637A>G	.	.	.	.	.	0%	0.09%
TARDBP	1	11085433	UTR3	c.*2722A>G	.	.	.	.	.	0%	0.09%
TARDBP	1	11085509	UTR3	c.*2798T>A	.	.	.	.	.	0.16%	0%
DCTN1	2	74588769	intronic	G>T	.	0.000154	.	1.63E-05	.	0%	0.09%
DCTN1	2	74590564	intronic	C>T	0.0005	0.000231	.	9.76E-05	rs201516838	0.33%	0%
DCTN1	2	74590583	intronic	C>A	.	.	.	.	.	0%	0.09%
DCTN1	2	74596051	intronic	G>C	.	.	.	.	.	0%	0.09%
ALS2	2	202571747	splicing	c.4404-2A>G	.	.	.	.	.	0%	0.09%
ALS2	2	202587758	intronic	T>-	.	.	.	.	.	0%	0.09%
ALS2	2	202588047	intronic	A>G	.	.	.	.	.	0%	0.18%
ALS2	2	202591597	intronic	C>G	.	.	.	.	.	0%	0.09%
ALS2	2	202598195	intronic	A>T	.	.	.	.	.	0.16%	0%
ALS2	2	202619414	intronic	G>-	.	0.002535	.	1.99E-03	.	0%	0.09%
ALS2	2	202622081	intronic	G>A	0.0005	.	.	7.35E-05	rs183864580	0%	0.09%
ALS2	2	202622501	intronic	A>C	0.0005	.	.	3.71E-04	rs200448709	0%	0.09%
ALS2	2	202632123	intronic	C>T	.	.	.	.	.	0.16%	0%
CHMP2B	3	87276547	UTR5	c.-126C>T	.	.	.	.	.	0%	0.09%
CHMP2B	3	87276666	UTR5	c.-7T>C	.	.	.	.	.	0%	0.09%
CHMP2B	3	87276714	intronic	C>T	0.0023	0.00469	.	2.77E-03	rs35413339	0.33%	0%
CHMP2B	3	87295096	intronic	T>A	.	.	.	8.15E-06	.	0.16%	0%
CHMP2B	3	87302682	intronic	A>G	.	0.000077	.	1.71E-04	.	0.33%	0.09%
CHMP2B	3	87303002	UTR3	c.*30T>G	.	.	.	.	.	0%	0.09%
CHMP2B	3	87303086	UTR3	c.*114C>T	.	.	.	.	.	0%	0.09%
CHMP2B	3	87303107	UTR3	c.*135T>C	.	.	.	.	.	0%	0.18%
SQSTM1	5	179251145	intronic	C>T	0.0005	0.001076	.	6.59E-04	rs139142649	0%	0.27%
SQSTM1	5	179251365	intronic	G>A	.	.	.	.	.	0.33%	0%
SQSTM1	5	179252235	intronic	A>T	.	.	.	8.13E-06	.	0%	0.09%
SQSTM1	5	179252237	intronic	C>T	.	.	.	8.13E-06	.	0%	0.09%
SQSTM1	5	179263768	UTR3	c.*175T>G	0.0009	.	.	.	rs186996560	0.33%	0.36%
TREM2	6	41129368	intronic	C>T	.	.	.	.	.	0%	0.09%
FIG4	6	110081590	intronic	A>T	.	.	.	.	.	0%	0.09%
FIG4	6	110081631	intronic	G>A	.	0.000461	.	3.42E-04	.	0.33%	0.44%
VCP	9	35056414	UTR3	c.*700C>A	.	.	.	.	.	0%	0.09%
VCP	9	35056530	UTR3	c.*584T>C	.	.	.	.	.	0.33%	0.18%
VCP	9	35056604	UTR3	c.*510A>G	.	.	.	.	.	0.16%	0%
VCP	9	35056818	UTR3	c.*296T>C	.	.	.	.	.	0%	0.09%
VCP	9	35056862	UTR3	c.*252C>T	.	.	.	.	.	0%	0.09%
VCP	9	35056898	UTR3	c.*216G>A	.	.	.	.	.	0%	0.44%
VCP	9	35057102	UTR3	c.*12C>T	0.0005	0.000923	.	1.08E-03	rs62544156	0.16%	0%
VCP	9	35057110	UTR3	c.*4G>T	.	0.000384	.	6.67E-04	rs201091341	0%	0.09%
VCP	9	35057227	intronic	C>T	.	.	.	.	.	0.16%	0%
VCP	9	35057267	intronic	G>A	.	0.000769	.	3.25E-04	.	0.49%	0.36%
VCP	9	35057558	intronic	T>C	.	.	.	.	.	0.82%	0.89%
VCP	9	35059029	intronic	T>C	0.0009	0.002768	.	3.39E-03	rs201932327	0%	0.09%
VCP	9	35059032	intronic	G>A	.	.	.	.	.	0%	0.09%
VCP	9	35059455	intronic	T>C	.	.	.	.	.	0%	0.09%
VCP	9	35060542	intronic	G>A	.	.	.	.	.	0%	0.09%
VCP	9	35060757	intronic	T>C	.	0.000077	.	4.07E-05	.	0.16%	0%
VCP	9	35060763	intronic	G>A	.	.	.	1.63E-05	.	0%	0.09%
VCP	9	35060765	intronic	G>A	.	.	.	.	.	0.82%	0%
VCP	9	35060781	intronic	G>T	.	.	.	.	.	0%	0.09%
VCP	9	35060934	intronic	G>C	0.0018	0.004921	.	3.00E-03	rs144304208	0%	0.09%
VCP	9	35060942	intronic	G>A	.	.	.	.	.	0%	0.36%
VCP	9	35061695	intronic	C>A	0.0018	.	.	1.78E-03	rs12349922	0%	0.18%
VCP	9	35061973	intronic	G>A	.	.	.	2.21E-03	.	0%	0.09%
VCP	9	35061983	intronic	G>A	0.0005	0.001	.	1.62E-03	rs200756991	0.33%	0.09%
VCP	9	35062381	intronic	T>C	.	.	.	.	.	0%	0.09%
VCP	9	35064324	intronic	T>C	0.0005	0.002153	.	1.39E-03	rs200165441	0%	0.09%
VCP	9	35067865	intronic	G>A	.	.	.	8.13E-06	.	0%	0.09%
VCP	9	35068102	intronic	G>A	0.0009	0.002076	.	1.63E-03	rs183916253	0%	0.09%
VCP	9	35068378	intronic	G>T	.	.	.	.	.	0%	0.09%
VCP	9	35068403	intronic	G>A	.	0.000308	.	7.32E-05	.	0%	0.09%
VCP	9	35072375	UTR5	c.-25C>T	.	.	.	.	.	0%	0.09%
VCP	9	35072378	UTR5	c.-28C>T	.	.	.	.	.	0.16%	0%
VCP	9	35072565	UTR5	c.-215A>G	.	.	.	.	.	0%	0.09%
VCP	9	35072576	UTR5	c.-226C>G	.	.	.	.	.	0%	0.09%
VCP	9	35072577	UTR5	c.-227G>C	.	.	.	.	.	0%	0.09%
VCP	9	35072657	UTR5	c.-307A>G	.	.	.	.	.	0.33%	0.36%
VCP	9	35072665	UTR5	c.-315G>A	.	.	.	.	.	0.16%	0%
VCP	9	35072710	UTR5	c.-360G>A	.	.	.	.	.	0.33%	0.27%
VCP	9	35072718	UTR5	c.-368A>T	.	.	.	.	.	0.33%	0%
VCP	9	35072736	UTR5	c.-386A>G	.	.	.	.	.	0.33%	0.09%
VCP	9	35072774	upstream	C>A	.	.	.	.	.	0%	0.09%
VCP	9	35072778	upstream	T>C	.	.	.	.	.	0%	0.09%
SETX	9	135171462	intronic	G>A	.	.	.	.	.	0%	0.09%
SETX	9	135205923	intronic	G>A	.	.	.	.	.	0.16%	0.80%
OPTN	10	13150099	intronic	G>C	.	.	.	.	.	0%	0.18%
OPTN	10	13150190	UTR5	c.-933G>A	.	.	.	.	.	0%	0.09%

OPTN	10	13150288	UTR5	c.-835T>C	.	.	.	.	.	0%	0.09%
OPTN	10	13151307	intronic	T>C	.	.	.	.	.	0%	0.09%
OPTN	10	13151314	intronic	C>T	.	0.000308	.	3.58E-04	rs200525334	0.16%	0.62%
OPTN	10	13151318	intronic	T>C	.	.	.	.	.	0%	0.09%
OPTN	10	13158341	splicing	c.626+1G>T	.	.	.	.	.	0%	0.09%
OPTN	10	13158389	intronic	C>G	.	.	.	.	.	0%	0.09%
OPTN	10	13167373	intronic	G>A	.	0.000154	.	4.07E-05	.	0%	0.09%
OPTN	10	13167594	intronic	C>T	0.0014	0.001845	.	1.46E-03	rs184547000	0.16%	0%
OPTN	10	13169905	splicing	c.1401+2T>G	.	.	.	.	.	0%	0.09%
OPTN	10	13175480	intronic	G>A	.	.	.	4.07E-05	.	0%	0.09%
OPTN	10	13175591	intronic	G>A	0.0032	0.000461	.	1.31E-03	rs191671333	0%	0.09%
OPTN	10	13178740	intronic	C>T	.	.	.	8.13E-06	.	0.33%	0.18%
OPTN	10	13178925	UTR3	c.*59G>C	.	.	.	.	.	0.33%	0%
OPTN	10	13179498	UTR3	c.*632G>C	.	.	.	.	.	0.16%	0%
OPTN	10	13179540	UTR3	c.*674A>T	.	.	.	.	.	0%	0.09%
OPTN	10	13179636	UTR3	c.*770delA	.	.	.	.	.	0%	0.09%
OPTN	10	13179749	UTR3	c.*883delA	.	.	.	.	.	0%	0.09%
OPTN	10	13179757	UTR3	c.*891T>C	.	.	.	.	.	0%	0.09%
OPTN	10	13179764	UTR3	c.*898C>T	.	.	.	.	.	0.33%	0.09%
OPTN	10	13179854	UTR3	c.*988C>G	.	.	.	.	.	0%	0.09%
OPTN	10	13179929	UTR3	c.*1063C>A	.	.	.	.	.	0.33%	0%
OPTN	10	13179955	UTR3	c.*1089G>A	.	.	.	.	.	0%	0.09%
OPTN	10	13180117	UTR3	c.*1251C>G	.	.	.	.	.	0%	0.09%
OPTN	10	13180149	UTR3	c.*1283C>T	.	.	.	.	.	0.16%	0%
OPTN	10	13180151	UTR3	c.*1285T>C	.	.	0.007	.	.	0%	0.27%
ANG	14	21162248	UTR3	c.*81A>C	.	.	0.007	.	.	0%	0.09%
FUS	16	31191566	intronic	G>A	.	.	.	.	.	0.49%	0.62%
FUS	16	31193691	intronic	A>T	.	0.000231	.	1.38E-04	.	0.16%	0%
FUS	16	31195147	intronic	C>G	.	0.000616	.	3.01E-04	.	0%	0.09%
FUS	16	31196244	intronic	->T	.	.	.	.	.	0%	0.09%
FUS	16	31198098	intronic	C>T	.	0.00077	.	5.53E-04	rs200247599	0%	0.09%
FUS	16	31198170	intronic	A>T	.	0.000077	.	3.25E-05	.	0.16%	0.09%
FUS	16	31198195	intronic	C>A	.	.	.	.	.	0%	0.09%
FUS	16	31199620	intronic	T>G	.	.	.	8.13E-06	.	0%	0.09%
FUS	16	31199626	intronic	T>G	.	.	.	8.13E-06	.	0.16%	0%
FUS	16	31199724	intronic	C>G	.	0.000693	.	7.08E-04	rs201544187	0.16%	0%
FUS	16	31199725	intronic	A>C	.	0.000154	.	2.11E-04	.	0.33%	0%
FUS	16	31201158	intronic	G>A	0.0005	0.000462	.	4.96E-04	rs200156095	0.33%	0.18%
FUS	16	31201159	intronic	G>C	.	.	.	8.13E-06	.	0.16%	0.09%
FUS	16	31201314	intronic	A>T	.	.	.	3.10E-04	.	0%	0.09%
FUS	16	31201316	intronic	T>C	.	.	.	1.06E-04	.	0%	0.09%
FUS	16	31201325	intronic	C>T	0.0005	0.000231	.	1.96E-04	rs181287335	0%	0.09%
FUS	16	31201335	intronic	C>T	0.0005	.	.	1.47E-04	rs185933137	0.16%	0.09%
FUS	16	31202019	intronic	C>T	.	.	.	8.13E-06	.	0%	0.09%
FUS	16	31202028	intronic	A>G	.	0.001	.	9.27E-04	rs200554137	0%	0.09%
FUS	16	31202048	intronic	T>C	.	.	.	.	.	0%	0.36%
FUS	16	31202168	intronic	G>A	.	.	.	.	.	0%	0.09%
FUS	16	31202268	intronic	G>C	.	.	.	.	.	0%	0.09%
FUS	16	31202674	intronic	T>G	.	.	.	.	rs74558144	0.33%	0.18%
FUS	16	31202777	UTR3	c.*18G>C	.	.	.	.	.	0.16%	0.09%
FUS	16	31202875	UTR3	c.*116G>A	.	.	.	8.25E-06	.	0.49%	0%
FUS	16	31202891	UTR3	c.*132C>A	.	.	.	1.27E-04	.	0%	0.09%
FUS	16	31202966	UTR3	c.*207G>A	.	.	.	1.66E-05	.	0%	0.18%
FUS	16	31202973	UTR3	c.*214C>T	0.0032	.	0.007	1.55E-03	rs140875749	0%	0.09%
FUS	16	31202990	UTR3	c.*231A>C	.	.	.	2.13E-05	.	0%	0.09%
FUS	16	31203167	UTR3	c.*408A>G	.	.	.	.	.	0.49%	0.53%
FUS	16	31203200	UTR3	c.*441A>G	.	.	.	.	.	0%	0.09%
FUS	16	31203320	UTR3	c.*561C>T	0.0009	.	.	1.82E-04	rs192705444	0.16%	0%
FUS	16	31203480	UTR3	c.*721A>G	.	.	.	1.19E-04	.	0%	0.09%
FUS	16	31203617	UTR3	c.*858G>T	.	.	.	.	.	0%	0.09%
FUS	16	31204538	UTR3	c.*1779T>C	.	.	.	.	.	0%	0.09%
FUS	16	31204593	UTR3	c.*1834A>G	.	.	.	.	.	0%	0.09%
FUS	16	31204602	UTR3	c.*1843G>A	.	.	.	6.23E-05	.	0%	0.09%
FUS	16	31204757	UTR3	c.*1998T>C	0.0032	.	0.007	4.71E-04	rs182437252	0%	0.09%
FUS	16	31204764	UTR3	c.*2005G>T	0.0018	.	.	4.10E-04	rs140979886	0%	0.09%
PFN1	17	4848916	downstream	T>G	0.0005	.	.	.	rs186959375	1.80%	1.07%
PFN1	17	4848931	downstream	C>T	.	.	.	.	.	0%	0.09%
PFN1	17	4848936	downstream	C>T	.	.	.	.	.	0%	0.09%
PFN1	17	4848948	UTR3	c.*247A>G	0.0023	.	.	.	rs140057352	0.16%	0%
PFN1	17	4848995	UTR3	c.*200T>C	.	.	.	.	.	0%	0.09%
PFN1	17	4849014	UTR3	c.*181G>A	.	.	.	.	.	0.33%	0.09%
PFN1	17	4849075	UTR3	c.*120C>T	0.0009	.	.	.	rs2233658	0%	0.09%
PFN1	17	4849087	UTR3	c.*108T>C	.	.	.	.	rs113941832	0%	0.09%
PFN1	17	4849123	UTR3	c.*72C>A	.	.	.	.	.	0%	0.09%
PFN1	17	4849169	UTR3	c.*26G>A	.	0.000154	.	6.67E-04	.	0.33%	0%
PFN1	17	4851695	UTR5	c.-6A>G	.	0.000077	.	2.44E-05	.	0%	0.09%
PFN1	17	4851767	UTR5	c.-78C>T	.	.	.	4.14E-03	.	0.16%	0%
VAPB	20	56993448	intronic	G>T	.	.	.	.	.	0.16%	0%
VAPB	20	56993466	intronic	T>C	.	.	.	.	.	0.33%	0%
SOD1	21	33031973	UTR5	c.-110T>C	.	.	.	.	.	0%	0.09%
SOD1	21	33031996	UTR5	c.-87G>A	.	.	.	.	.	1.15%	0.44%
SOD1	21	33032173	intronic	G>A	.	0.000077	.	4.32E-04	rs201574089	0%	0.09%
SOD1	21	33032189	intronic	G>-	.	.	.	.	.	0.33%	0%
SOD1	21	33032202	intronic	C>T	.	.	.	1.14E-04	.	0%	0.09%
SOD1	21	33032205	intronic	C>G	.	0.000231	.	3.51E-04	.	0%	0.09%
SOD1	21	33036099	intronic	A>G	.	0.000231	.	8.95E-05	.	0%	0.18%
SOD1	21	33038757	intronic	A>C	.	.	.	.	.	0.33%	0%
SOD1	21	33038863	intronic	C>T	.	.	.	8.13E-06	.	0.33%	0.36%
SOD1	21	33040970	UTR3	c.*79T>A	.	.	.	.	.	0.98%	1.60%
SOD1	21	33040981	UTR3	c.*90A>T	.	.	.	.	.	0%	0.09%
SOD1	21	33041012	UTR3	c.*121A>G	.	.	.	.	.	0.33%	0%
SOD1	21	33041044	UTR3	c.*153T>G	0.0005	.	.	.	rs188029963	0.33%	0%
SOD1	21	33041092	UTR3	c.*201A>G	.	.	.	.	.	0%	0.09%
SOD1	21	33041143	UTR3	c.*252G>T	.	.	.	.	.	0%	0.09%
SOD1	21	33041171	UTR3	c.*280A>G	.	.	.	.	.	0.33%	0%
SOD1	21	33041263	downstream	T>A	0.0014	.	.	.	rs184438684	0%	0.09%
UBQLN2	X	56589985	upstream	C>T	.	.	.	.	.	0%	0.09%
UBQLN2	X	56590003	upstream	C>T	.	.	.	.	.	0%	0.09%
UBQLN2	X	56590263	UTR5	c.-44G>C	.	.	.	.	.	0%	0.09%
UBQLN2	X	56590303	UTR5	c.-4C>T	.	.	.	8.76E-06	.	0%	0.09%
UBQLN2	X	56592268	UTR3	c.*87A>C	.	.	.	.	.	0.33%	0%
UBQLN2	X	56592316	UTR3	c.*135A>G	.	.	.	.	.	0%	0.18%
UBQLN2	X	56592584	UTR3	c.*403C>A	.	.	.	.	.	0%	0.09%
UBQLN2	X	56592587	UTR3	c.*406G>A	.	.	.	.	.	0%	0.09%
UBQLN2	X	56592606	UTR3	c.*425C>T	.	.	.	.	.	0.33%	0.18%

UBQLN2	X	56592686	UTR3	c.*505T>G	.	.	.	.	.	0%	0.18%
UBQLN2	X	56592754	UTR3	c.*573A>G	.	.	.	.	.	0%	0.09%
UBQLN2	X	56592850	UTR3	c.*669A>G	.	.	.	.	.	0.16%	0%
UBQLN2	X	56593074	UTR3	c.*893C>A	.	.	.	.	.	0%	0.18%
UBQLN2	X	56593415	UTR3	c.*1234G>A	0.0018	.	.	.	rs187165435	0%	0.09%

Supplementary Table 6. List of patients with two variants of interest.

Subject	Variant1	Variant2	El-Escorial	Gender	Family history
1	TARDBP N179D	TARDBP M337V	ALS-Probable	Male	Sporadic
2	C9orf72 expansion	VCP R155H	ALS-Definite	Male	Sporadic
3	C9orf72 expansion	TARDBP A321V	ALS-Probable	Male	Familial
4	C9orf72 expansion	UBQLN2 T334M	ALS-Definite	Female	Sporadic
5	C9orf72 expansion	ALS2 S654G	ALS-Definite	Male	Familial
6	C9orf72 expansion	ANG K78E	ALS-Definite	Female	Familial
7	C9orf72 expansion	OPTN c.626+1G>T	ALS-Probable	Male	Sporadic
8	FUS S135N	SOD1 D77Y	ALS-Definite	Male	Familial
9	OPTN R271H	FUS R269W	ALS-Definite	Female	Familial
10	SOD1 D91A	UBQLN2 Q460R	ALS-Probable	Male	Familial
11	ALS2 P1288L	SOD1 I114T	ALS-Probable	Male	Familial
12	ALS2 P372R	TARDBP A90V	Control	Female	N/A

Supplementary Table 7. List of variants in *VEGFA* and *PON1-3*. All variants were sequenced adequately in controls however some were not adequately covered in cases. No variants were found to be significantly more in cases than controls after correction for multiple testing. Some other loci were also sequenced however they failed quality checks and so were removed, particularly in *VEGFA* due the high GC content.

Gene	Chromosome	Base pair	dbSNP137	Variant	Frequency Controls	Frequency Patient	Number of Patients Sequenced	P-value	ExAC	1000G
VEGFA	6	43748593	rs149528656	c.1085+2T>C	0.2%	0%	95	0.66	0.01%	NA
VEGFA	6	43748600	rs201132204	c.1085+9T>C	0.2%	0%	120	0.56	1%	NA
PON1	7	94937412	rs141624867	c.G609A:p.S203S	0.2%	0%	1013	0.60	0.01%	NA
PON1	7	94937418	rs148452713	c.G603A:p.A201A	0%	0.1%	1013	0.01	0.1%	0.2%
PON1	7	94937419	rs80019660	c.C602T:p.A201V	0.5%	0.5%	1013	1.00	0.2%	0.1%
PON1	7	94937446	rs662	c.A575G:p.Q192R	49%	50%	1108	0.78	38%	52%
PON1	7	94946084	rs854560	c.T163A:p.L55M	60%	61%	1108	0.89	29%	20%
PON1	7	94953733	rs141948033	c.A55G:p.N19D	0.4%	0%	132	0.54	0.2%	0.1%
PON1	7	94953881	.	c.-94C>T	0%	0.2%	1104	0.04	NA	NA
PON1	7	94953895	rs705379	c.-108C>T	71%	72%	1104	0.85	NA	38%
PON1	7	94953913	rs705380	c.-126C>G	99%	99%	1105	0.42	NA	95%
PON3	7	94993261	rs17880470	c.T609C:p.Y203Y	2%	0.8%	119	0.54	0.4%	0.3%
PON3	7	94993334	rs17883013	c.C536A:p.A179D	0%	1%	95	0.02	0.5%	1%
PON3	7	95001555	rs1053275	c.G297A:p.A99A	71%	71%	95	0.52	57%	63%
PON3	7	95001590	rs78883915	c.A262G:p.M88V	0.2%	0%	95	0.66	0.1%	0.1%
PON2	7	95034775	rs7493	c.C932G:p.S311C	43%	0%	95	0.50	27%	26%
PON2	7	95034821	rs9641164	c.907-21T>A	34%	42%	95	0.14	24%	30%
PON2	7	95041016	rs12026	c.C407G:p.A136G	43%	8%	95	0.75	27%	26%
PON2	7	95041135	rs17876141	c.368-44G>A	0%	1%	95	0.03	0.6%	2%
PON2	7	95041704	rs201552995	c.G287C:p.R96T	0.4%	1%	95	0.45	NA	NA

Supplementary Table 8. Variants included in Fig. 1 and the references and extra information associated with each.

Gene	Variant	No. of variants in patients	Total no. of patients	No. of variants in controls	Total no. of controls	Reference	Nationality
FUS	c.-54A>G	187	446	NA	NA	Brown *et al.*, 2012	United states
FUS	c.-2A>T	1	66	0	561	Yan *et al.*, 2010	Italian
FUS	c.*14C>T	2	220	0	151	Zou *et al.*, 2012	Caucasian
FUS	c.*41G>A	3	323	0	216	Brown *et al.*, 2012	United States

FUS	c.*41G>A	29	1009	17	538	Corrado *et al.*, 2010	Italian
FUS	c.*41G>A	2	70	9	569	Huey *et al.*, 2012	United states
FUS	c.*41G>A	1	94	0	376	Ticozzi *et al.*, 2009	Chinese
FUS	c.*41G>A	1	116	0	700	DeJesus-Hernandez *et al.*, 2010	Italian
FUS	c.*41G>A	20	420	17	480	Sabatelli *et al.*, 2013	Italian
FUS	c.*47C>T	1	116	0	700	De-Jesus-Harnandez 2010	Italian
FUS	C.-48G>A	1	420	0	480	Sabatelli *et al.*, 2013	Italian
FUS	C.*59G>A	1	420	0	480	Sabatelli *et al.*, 2013	Italian
FUS	C.*105dup	1	323	0	216	Brown *et al.*, 2012	United States
FUS	C.*108C>T	1	420	0	480	Sabatelli *et al.*, 2013	Italian
FUS	C.*110G>A	1	420	0	480	Sabatelli *et al.*, 2013	Italian
FUS	c.*132C>A	1	323	0	216	Brown *et al.*, 2012	United States
FUS	c.*190C>A	1	323	0	216	Brown *et al.*, 2012	United States
FUS	c.*214C>T	3	446	4	216	Brown *et al.*, 2012	United states
FUS	c.*214C>T	6	420	10	480	Sabatelli et al., 2013	Italian
TARDBP	c.*82A>G	0	177	1	200	Chiang *et al.*, 2012	Nordic
TARDBP	c.*620A>G	1	177	0	200	Chiang *et al.*, 2012	Nordic
TARDBP	c.*533C>G	0	177	1	200	Chiang *et al.*, 2012	Nordic
TARDBP	c.*343G>A	0	177	1	200	Chiang *et al.*, 2012	Nordic
TARDBP	c.*208G>A	16	177	18	200	Chiang *et al.*, 2012	Nordic
TARDBP	c.*2076G>A	2	38	0	982	Gitcho *et al.*, 2009	United States
TARDBP	c.*1622A>T	1	177	1	200	Chiang *et al.*, 2012	Nordic
TARDBP	c.*1462T>C	1	285	0	360	Daoud *et al.*, 2009	French
TARDBP	c.*1453G>A	6	149	4	100	Benajiba *et al.*, 2009	French
TARDBP	c.*1008T>G	1	177	1	200	Chiang *et al.*, 2012	Nordic

Supplemental references

Abramzon Y, Johnson JO, Scholz SW, Taylor JP, Brunetti M, Calvo A, et al. Valosin-containing protein (VCP) mutations in sporadic amyotrophic lateral sclerosis. Neurobiol. Aging 2012; 33: 2231.e1-2231.e6.

Andersen PM, Sims KB, Xin WW, Kiely R, O'Neill G, Ravits J, et al. Sixteen novel mutations in the Cu/Zn superoxide dismutase gene in amyotrophic lateral sclerosis: a decade of discoveries, defects and disputes. Amyotroph. Lateral Scler. Other Motor Neuron Disord. 2003; 4: 62–73.

Arning L, Epplen JT, Rahikkala E, Hendrich C, Ludolph AC, Sperfeld A-D. The SETX missense variation spectrum as evaluated in patients with ALS4-like motor neuron diseases. neurogenetics 2012; 14: 53–61.

Ayers J, Lelie H, Workman A, Prudencio M, Brown H, Fromholt S, et al. Distinctive features of the D101N and D101G variants of superoxide dismutase 1; two mutations that produce rapidly progressing motor neuron disease. J. Neurochem. 2014; 128: 305–314.

Beck J, Pittman A, Adamson G, Campbell T, Kenny J, Houlden H, et al. Validation of next-generation sequencing technologies in genetic diagnosis of dementia. Neurobiol. Aging 2014; 35: 261–265.

Benajiba L, Le Ber I, Camuzat A, Lacoste M, Thomas-Anterion C, Couratier P, et al. TARDBP mutations in motoneuron disease with frontotemporal lobar degeneration. Ann. Neurol. 2009; 65: 470–473.

Blair IP, Williams KL, Warraich ST, Durnall JC, Thoeng AD, Manavis J, et al. FUS mutations in amyotrophic lateral sclerosis: clinical, pathological, neurophysiological and genetic analysis. J. Neurol. Neurosurg. Psychiatry 2010; 81: 639–645.

van Blitterswijk M, van Es MA, Hennekam EAM, Dooijes D, van Rheenen W, Medic J, et al. Evidence for an oligogenic basis of amyotrophic lateral sclerosis. Hum. Mol. Genet. 2012; 21: 3776–3784.

van Blitterswijk M, van Es MA, Koppers M, van Rheenen W, Medic J, Schelhaas HJ, et al. VAPB and C9orf72 mutations in 1 familial amyotrophic lateral sclerosis patient. Neurobiol. Aging 2012; 33: 2950.e1-4.

van Blitterswijk M, Vlam L, van Es MA, van der Pol W-L, Hennekam EAM, Dooijes D, et al. Genetic overlap between apparently sporadic motor neuron diseases. PloS One 2012; 7: e48983.

Brown JA, Min J, Staropoli JF, Collin E, Bi S, Feng X, et al. SOD1, ANG, TARDBP and FUS mutations in amyotrophic lateral sclerosis: a United States clinical testing lab experience. Amyotroph. Lateral Scler. Off. Publ. World Fed. Neurol. Res. Group Mot. Neuron Dis. 2012; 13: 217–222.

Buentello-Volante B, Elizondo-Olascoaga C, Miranda-Duarte A, Guadarrama-Vallejo D, Cabral-Macias J, Zenteno JC. Association study of multiple gene polymorphisms with the risk of adult-onset primary open-angle glaucoma in a Mexican population. Exp. Eye Res. 2013; 107: 59–64.

Bury JJ, Highley JR, Cooper-Knock J, Goodall EF, Higginbottom A, McDermott CJ, et al. Oligogenic inheritance of optineurin (OPTN) and C9ORF72 mutations in ALS highlights localisation of OPTN in the TDP-43-negative inclusions of C9ORF72-ALS. Neuropathol. Off. J. Jpn. Soc. Neuropathol. 2016; 36: 125–134.

Cady J, Allred P, Bali T, Pestronk A, Goate A, Miller TM, et al. Amyotrophic lateral sclerosis onset is influenced by the burden of rare variants in known amyotrophic lateral sclerosis genes. Ann. Neurol. 2015; 77: 100–113.

Cady J, Koval ED, Benitez BA, Zaidman C, Jockel-Balsarotti J, Allred P, et al. TREM2 variant p.R47H as a risk factor for sporadic amyotrophic lateral sclerosis. JAMA Neurol. 2014; 71: 449–453.

Chiang H-H, Andersen PM, Tysnes O-B, Gredal O, Christensen PB, Graff C. Novel TARDBP mutations in Nordic ALS patients. J. Hum. Genet. 2012; 57: 316–319.

Chow CY, Zhang Y, Dowling JJ, Jin N, Adamska M, Shiga K, et al. Mutation of FIG4 causes neurodegeneration in the pale tremor mouse and patients with CMT4J. Nature 2007; 448: 68–72.

Conte A, Lattante S, Luigetti M, Grande AD, Romano A, Marcaccio A, et al. Classification of familial amyotrophic lateral sclerosis by family history: effects on frequency of genes mutation. J. Neurol. Neurosurg. Psychiatry 2012: jnnp-2012-302897.

Corrado L, Bo RD, Castellotti B, Ratti A, Cereda C, Penco S, et al. Mutations of FUS gene in sporadic amyotrophic lateral sclerosis. J. Med. Genet. 2010; 47: 190–194.

Corrado L, Carlomagno Y, Falasco L, Mellone S, Godi M, Cova E, et al. A novel peripherin gene (PRPH) mutation identified in one sporadic amyotrophic lateral sclerosis patient. Neurobiol. Aging 2011; 32: 552.e1-552.e6.

Covassin T. Free Communications, Oral Presentations: New Concepts in the Study of Sports-Related Concussion Dilemma. J. Athl. Train. 2010; 45: S29–S31.

Cox LE, Ferraiuolo L, Goodall EF, Heath PR, Higginbottom A, Mortiboys H, et al. Mutations in CHMP2B in Lower Motor Neuron Predominant Amyotrophic Lateral Sclerosis (ALS). PLOS ONE 2010; 5: e9872.

Daoud H, Valdmanis PN, Gros-Louis F, Belzil V, Spiegelman D, Henrion E, et al. Resequencing of 29 candidate genes in patients with familial and sporadic amyotrophic lateral sclerosis. Arch. Neurol. 2011; 68: 587–593.

Daoud H, Valdmanis PN, Kabashi E, Dion P, Dupré N, Camu W, et al. Contribution of TARDBP mutations to sporadic amyotrophic lateral sclerosis. J. Med. Genet. 2009; 46: 112–114.

DeJesus-Hernandez, Kocerha J, Finch N, Crook R, Baker M, Desaro P, et al. De novo truncating FUS gene mutation as a cause of sporadic amyotrophic lateral sclerosis. Hum. Mutat. 2010; 31: E1377–E1389.

Del Bo R, Tiloca C, Pensato V, Corrado L, Ratti A, Ticozzi N, et al. Novel optineurin mutations in patients with familial and sporadic amyotrophic lateral sclerosis. J. Neurol. Neurosurg. Psychiatry 2011; 82: 1239–1243.

Deng H-X, Chen W, Hong S-T, Boycott KM, Gorrie GH, Siddique N, et al. Mutations in UBQLN2 cause dominant X-linked juvenile and adult-onset ALS and ALS/dementia. Nature 2011; 477: 211–215.

Deng H-X, Zhai H, Bigio EH, Yan J, Fecto F, Ajroud K, et al. FUS-immunoreactive inclusions are a common feature in sporadic and non-SOD1 familial amyotrophic lateral sclerosis. Ann. Neurol. 2010; 67: 739–748.

Drepper C, Herrmann T, Wessig C, Beck M, Sendtner M. C-terminal FUS/TLS mutations in familial and sporadic ALS in Germany. Neurobiol. Aging 2011; 32: 548.e1-4.

Eisen A, Mezei MM, Stewart HG, Fabros M, Gibson G, Andersen PM. SOD1 gene mutations in ALS patients from British Columbia, Canada: clinical features, neurophysiology and ethical issues in management. Amyotroph. Lateral Scler. Off. Publ. World Fed. Neurol. Res. Group Mot. Neuron Dis. 2008; 9: 108–119.

Fecto F, Yan J, Vemula SP, Liu E, Yang Y, Chen W, et al. SQSTM1 mutations in familial and sporadic amyotrophic lateral sclerosis. Arch. Neurol. 2011; 68: 1440–1446.

Figlewicz DA, Krizus A, Martinoli MG, Meininger V, Dib M, Rouleau GA, et al. Variants of the heavy neurofilament subunit are associated with the development of amyotrophic lateral sclerosis. Hum. Mol. Genet. 1994; 3: 1757–1761.

Fratta P, Charnock J, Collins T, Devoy A, Howard R, Malaspina A, et al. Profilin1 E117G is a moderate risk factor for amyotrophic lateral sclerosis. J. Neurol. Neurosurg. Psychiatry 2014; 85: 506–508.

Gijselinck I, Sleegers K, Engelborghs S, Robberecht W, Martin J-J, Vandenberghe R, et al. Neuronal inclusion protein TDP-43 has no primary genetic role in FTD and ALS. Neurobiol. Aging 2009; 30: 1329–1331.

Gitcho MA, Bigio EH, Mishra M, Johnson N, Weintraub S, Mesulam M, et al. TARDBP 3'-UTR variant in autopsy-confirmed frontotemporal lobar degeneration with TDP-43 proteinopathy. Acta Neuropathol. (Berl.) 2009; 118: 633–645.

Gonzalez MA, Feely SM, Speziani F, Strickland AV, Danzi M, Bacon C, et al. A novel mutation in VCP causes Charcot-Marie-Tooth Type 2 disease. Brain J. Neurol. 2014; 137: 2897–2902.

Greenway MJ, Andersen PM, Russ C, Ennis S, Cashman S, Donaghy C, et al. ANG mutations segregate with familial and 'sporadic' amyotrophic lateral sclerosis. Nat. Genet. 2006; 38: 411–413.

Groen EJN, van Es MA, van Vught PWJ, Spliet WGM, van Engelen-Lee J, de Visser M, et al. FUS mutations in familial amyotrophic lateral sclerosis in the Netherlands. Arch. Neurol. 2010; 67: 224–230.

Gros-Louis F, Larivière R, Gowing G, Laurent S, Camu W, Bouchard J-P, et al. A Frameshift Deletion in Peripherin Gene Associated with Amyotrophic Lateral Sclerosis. J. Biol. Chem. 2004; 279: 45951–45956.

Guerreiro RJ, Lohmann E, Brás JM, Gibbs JR, Rohrer JD, Gurunlian N, et al. Using exome sequencing to reveal mutations in TREM2 presenting as a frontotemporal dementia-like syndrome without bone involvement. JAMA Neurol. 2013; 70: 78–84.

Guerreiro RJ, Schymick JC, Crews C, Singleton A, Hardy J, Traynor BJ. TDP-43 Is Not a Common Cause of Sporadic Amyotrophic Lateral Sclerosis. PLOS ONE 2008; 3: e2450.

Hand CK, Devon RS, Gros-Louis F, Rochefort D, Khoris J, Meininger V, et al. Mutation screening of the ALS2 gene in sporadic and familial amyotrophic lateral sclerosis. Arch. Neurol. 2003; 60: 1768–1771.

Herzfeld T, Wolf N, Winter P, Hackstein H, Vater D, Müller U. Maternal uniparental heterodisomy with partial isodisomy of a chromosome 2 carrying a splice acceptor site mutation (IVS9-2A>T) in ALS2 causes infantile-onset ascending spastic paralysis (IAHSP). Neurogenetics 2009; 10: 59–64.

Hineno A, Nakamura A, Shimojima Y, Yoshida K, Oyanagai K, Ikeda S. Distinctive clinicopathological features of 2 large families with amyotrophic lateral sclerosis having L106V mutation in SOD1 gene. J. Neurol. Sci. 2012; 319: 63–74.

Huey ED, Ferrari R, Moreno JH, Jensen C, Morris CM, Potocnik F, et al. FUS and TDP43 genetic variability in FTD and CBS. Neurobiol. Aging 2012; 33: 1016.e9-1016.17.

Kabashi E, Valdmanis PN, Dion P, Spiegelman D, McConkey BJ, Vande Velde C, et al. TARDBP mutations in individuals with sporadic and familial amyotrophic lateral sclerosis. Nat. Genet. 2008; 40: 572–574.

Kirby J, Goodall EF, Smith W, Highley JR, Masanzu R, Hartley JA, et al. Broad clinical phenotypes associated with TAR-DNA binding protein (TARDBP) mutations in amyotrophic lateral sclerosis. Neurogenetics 2010; 11: 217–225.

Kokubo Y, Kuzuhara S, Narita Y, et al. Accumulation of neurofilaments and sod1-immunoreactive products in a patient with familial amyotrophic lateral sclerosis with i113t sod1 mutation. Arch. Neurol. 1999; 56: 1506–1508.

Kun-Rodrigues C, Ganos C, Guerreiro R, Schneider SA, Schulte C, Lesage S, et al. A systematic screening to identify de novo mutations causing sporadic early-onset Parkinson's disease. Hum. Mol. Genet. 2015; 24: 6711–6720.

Lattante S, Le Ber I, Camuzat A, Dayan S, Godard C, Van Bortel I, et al. TREM2 mutations are rare in a French cohort of patients with frontotemporal dementia. Neurobiol. Aging 2013; 34: 2443.e1-2.

Lenk GM, Ferguson CJ, Chow CY, Jin N, Jones JM, Grant AE, et al. Pathogenic mechanism of the FIG4 mutation responsible for Charcot-Marie-Tooth disease CMT4J. PLoS Genet. 2011; 7: e1002104.

Lopate G, Baloh RH, Al-Lozi MT, Miller TM, Fernandes Filho JA, Ni O, et al. Familial ALS with extreme phenotypic variability due to the I113T SOD1 mutation. Amyotroph. Lateral Scler. Off. Publ. World Fed. Neurol. Res. Group Mot. Neuron Dis. 2010; 11: 232–236.

Mukhopadhyay A, Komatireddy S, Acharya M, Bhattacharjee A, Mandal AK, Thakur SKD, et al. Evaluation of Optineurin as a candidate gene in Indian patients with primary open angle glaucoma. Mol. Vis. 2005; 11: 792–797.

Münch C, Sedlmeier R, Meyer T, Homberg V, Sperfeld AD, Kurt A, et al. Point mutations of the p150 subunit of dynactin (DCTN1) gene in ALS. Neurology 2004; 63: 724–726.

Nakamura A, Hineno A, Yoshida K, Sekijima Y, Hanaoka-Tachibana N, Takei Y-I, et al. Marked intrafamilial phenotypic variation in a family with SOD1 C111Y mutation. Amyotroph. Lateral Scler. Off. Publ. World Fed. Neurol. Res. Group Mot. Neuron Dis. 2012; 13: 479–486.

Nanetti L, Cavalieri S, Pensato V, Erbetta A, Pareyson D, Panzeri M, et al. SETX mutations are a frequent genetic cause of juvenile and adult onset cerebellar ataxia with neuropathy and elevated serum alpha-fetoprotein. Orphanet J. Rare Dis. 2013; 8: 123.

Orrell RW, Habgood JJ, Malaspina A, Mitchell J, Greenwood J, Lane RJ, et al. Clinical characteristics of SOD1 gene mutations in UK families with ALS. J. Neurol. Sci. 1999; 169: 56–60.

Parkinson N, Ince PG, Smith MO, Highley R, Skibinski G, Andersen PM, et al. ALS phenotypes with mutations in CHMP2B (charged multivesicular body protein 2B). Neurology 2006; 67: 1074–1077.

Rajput A, Rajput AH, Rajput ML, Encarnacion M, Bernales CQ, Ross JP, et al. Identification of FUS p.R377W in essential tremor. Eur. J. Neurol. 2014; 21: 361–363.

Rayaprolu S, Mullen B, Baker M, Lynch T, Finger E, Seeley WW, et al. TREM2 in neurodegeneration: evidence for association of the p.R47H variant with frontotemporal dementia and Parkinson's disease. Mol. Neurodegener. 2013; 8: 19.

Rezaie T, Child A, Hitchings R, Brice G, Miller L, Coca-Prados M, et al. Adult-Onset Primary Open-Angle Glaucoma Caused by Mutations in Optineurin. Science 2002; 295: 1077–1079.

Robberecht W, Aguirre T, Bosch LVD, Tilkin P, Cassiman JJ, Matthijs G. D90A heterozygosity in the SOD1 gene is associated with familial and apparently sporadic amyotrophic lateral sclerosis. Neurology 1996; 47: 1336–1339.

Rohrer JD, Warren JD, Reiman D, Uphill J, Beck J, Collinge J, et al. A novel exon 2 I27V VCP variant is associated with dissimilar clinical syndromes. J. Neurol. 2011; 258: 1494–1496.

Rubino E, Rainero I, Chiò A, Rogaeva E, Galimberti D, Fenoglio P, et al. SQSTM1 mutations in frontotemporal lobar degeneration and amyotrophic lateral sclerosis. Neurology 2012; 79: 1556–1562.

Rudnik-Schöneborn S, Arning L, Epplen JT, Zerres K. SETX gene mutation in a family diagnosed autosomal dominant proximal spinal muscular atrophy. Neuromuscul. Disord. 2012; 22: 258–262.

Rutherford NJ, Zhang Y-J, Baker M, Gass JM, Finch NA, Xu Y-F, et al. Novel mutations in TARDBP (TDP-43) in patients with familial amyotrophic lateral sclerosis. PLoS Genet. 2008; 4: e1000193.

Sabatelli M, Moncada A, Conte A, Lattante S, Marangi G, Luigetti M, et al. Mutations in the 3' untranslated region of FUS causing FUS overexpression are associated with amyotrophic lateral sclerosis. Hum. Mol. Genet. 2013; 22: 4748–4755.

Soong B-W, Lin K-P, Guo Y-C, Lin C-CK, Tsai P-C, Liao Y-C, et al. Extensive molecular genetic survey of Taiwanese patients with amyotrophic lateral sclerosis. Neurobiol. Aging 2014; 35: 2423.e1-6.

Sreedharan J, Blair IP, Tripathi VB, Hu X, Vance C, Rogelj B, et al. TDP-43 mutations in familial and sporadic amyotrophic lateral sclerosis. Science 2008; 319: 1668–1672.

Suzuki N, Aoki M, Warita H, Kato M, Mizuno H, Shimakura N, et al. FALS with FUS mutation in Japan, with early onset, rapid progress and basophilic inclusion. J. Hum. Genet. 2010; 55: 252–254.

Swarup G, Vaibhava V, Nagabhush A. Functional Defects Caused by Glaucoma – Associated Mutations in Optineurin [Internet]. In: Rumelt S, editor(s). Glaucoma - Basic and Clinical Aspects. InTech; 2013. [cited 2016 Mar 21] Available from: http://www.intechopen.com/books/glaucoma-basic-and-clinical-aspects/functional-defects-caused-by-glaucoma-associated-mutations-in-optineurin

Tateishi T, Hokonohara T, Yamasaki R, Miura S, Kikuchi H, Iwaki A, et al. Multiple system degeneration with basophilic inclusions in Japanese ALS patients with FUS mutation. Acta Neuropathol. (Berl.) 2010; 119: 355–364.

Ticozzi N, Silani V, LeClerc AL, Keagle P, Gellera C, Ratti A, et al. Analysis of FUS gene mutation in familial amyotrophic lateral sclerosis within an Italian cohort. Neurology 2009; 73: 1180–1185.

Tsai C-P, Soong B-W, Lin K-P, Tu P-H, Lin J-L, Lee Y-C. FUS, TARDBP, and SOD1 mutations in a Taiwanese cohort with familial ALS. Neurobiol. Aging 2011; 32: 553.e13-553.e21.

Van Langenhove T, van der Zee J, Sleegers K, Engelborghs S, Vandenberghe R, Gijselinck I, et al. Genetic contribution of FUS to frontotemporal lobar degeneration. Neurology 2010; 74: 366–371.

Winton MJ, Van Deerlin VM, Kwong LK, Yuan W, Wood EM, Yu C-E, et al. A90V TDP-43 variant results in the aberrant localization of TDP-43 in vitro. FEBS Lett. 2008; 582: 2252–2256.

Wu C-H, Fallini C, Ticozzi N, Keagle PJ, Sapp PC, Piotrowska K, et al. Mutations in the profilin 1 gene cause familial amyotrophic lateral sclerosis. Nature 2012; 488: 499–503.

Yan J, Deng H-X, Siddique N, Fecto F, Chen W, Yang Y, et al. Frameshift and novel mutations in FUS in familial amyotrophic lateral sclerosis and ALS/dementia. Neurology 2010; 75: 807–814.

Zou Z-Y, Peng Y, Feng X-H, Wang X-N, Sun Q, Liu M-S, et al. Screening of the FUS gene in familial and sporadic amyotrophic lateral sclerosis patients of Chinese origin. Eur. J. Neurol. 2012; 19: 977–983.
